# Simulation of anaerobic biodegradation process in a tubular bioreactor with a biofilm layer: Steady-state and unsteady-state conditions

**DOI:** 10.1016/j.heliyon.2024.e35397

**Published:** 2024-07-30

**Authors:** Saman Malekahmadi, Amirhossein Yousefnezhadazizi, Hossein Askaripour

**Affiliations:** Department of Chemical and Petroleum Engineering, Sharif University of Technology, Tehran, Iran

**Keywords:** Tubular bioreactor, Biofilm layer, Anaerobic biodegradation, Proportional-integral controller, Steady- and unsteady-state simulation

## Abstract

In this paper, anaerobic biodegradation process in a tubular bioreactor with an inner biofilm layer for steady-state and unsteady-state conditions are simulated. The effects of various parameters including bioreactor diameter, fraction of active biomass transferred to liquid phase, and residence time of the liquid on bioreactor performance are examined. Simulations indicate that decreasing diameter of bioreactor leads to increasing degree of conversion of the substrate in liquid phase and decreasing dimensionless concentration of the substrate in biofilm. With an increase in the fraction of active biomass transferred to liquid, substrate concentrations in liquid and biofilm slightly vary. Increased residence time of the liquid phase results in the degree of conversion of substrate goes up, but substrate concentration in biofilm lowers a little. In addition, it is found that biomass concentration of liquid phase is boosted with decreased bioreactor diameter and increased residence time of liquid. A proportional-integral controller is designed and the tuned parameters of $K_{P}=-0.131$ and $K_{I}=0.02$ are obtained using genetic algorithm. It is observed that controller regulate well the degree of conversion of the substrate within 120 s for both servo and regulatory modes.

## Highlights

• Tubular bioreactor with biofilm is simulated for steady state and unsteady state.
• Parameters of proportional-integral controller are tuned using genetic algorithm.
• As Bioreactor diameter lowers, degree of conversion of substrate in liquid rises.
• Rising residence time leads to a small drop in substrate concentration in biofilm.
• Biomass concentration in liquid is boosted with decreased bioreactor diameter.

## Graphical abstract

**Figure:**
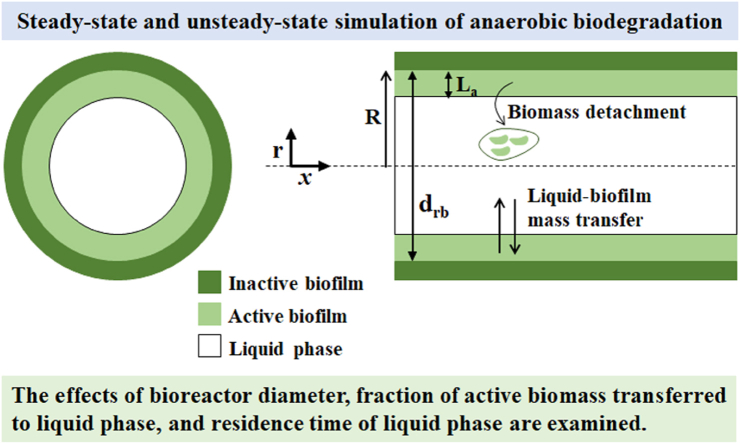

## 1 Introduction

In recent decades, new biological processes are emerged owing to the progress of biotechnology science, which differ in terms of process conditions including type of microorganisms and nutrients used in culture media, operational scale, hydrodynamics, and type and value of the products. It is apparent that diverse designs of bioreactors are needed for the successful operation of different bioprocesses. Despite the widespread use of continuous stirred tank bioreactors (CSTBR), other types of bioreactors, e.g., i.e., vertical, tubular, and horizontal ones, attract tremendous interest in the past years. This is because of their suitability for various processes including production of ethanol [1,2], pesticides [1], organic acids and biopolymers [3], wastewater treatment [4,5], and animal cell cultivation [6]. The industrial applications of bioreactors for chemicals production and microbiological processes as well as quality of the continuous production and high productivity increasingly result in development of high-performance bioreactor systems.

Phenol and their derivatives are widely utilized in many industrial processes such as petroleum refineries, tannery and foundries, pharmaceuticals, and pulp and paper mills. Because of toxicity and environmental threats of phenolic compounds, removing this chemical from industrial wastewater is essential. Several conventional methods like O_3_/UV, H_2_O_2_/UV, and Fenton processes, solvent extraction, and membrane separation have been applied in the literature. These physicochemical processes lead to some secondary problems for effluents, e.g., formation of chlorophenols as a result of chlorination of phenols. Biological removal of phenols is a preferable way to degrade these contaminants into other non-toxic compounds. It is one the main reasons why bioreactors have been widely employed for the phenol removal from wastewater of large-scale and industrial plants [7].

The use of tubular bioreactors for biochemical reactions with complicated kinetics including substrate and product inhibition can meet high productivity and conversion. Anaerobic process of ethanol production from glucose is an example of these problems, which was explored in the work of Moser [8]. Moser compared the conversion of CSTBR and tubular bioreactor and concluded that the latter is more advantageous. It is notable that continuous stirred tank bioreactors have some disadvantages like cell damage, high cost of cooling, and high power consumption [9]. Other types of bioreactors, e.g., tubular and airlift ones, were developed to overcome these shortcomings. The facile construction, less number of parameters needed for scale-up, more uniform mixing, high area-to-volume ratio, and more efficient heat and mass transfer are some of the advantages of tubular bioreactors [10,11].

Chang and Alvarez-Cohen [12] used a two-stage system including a CSTBR and a tubular bioreactor to treat the wastewater polluted by trichloroethylene and *cis*-l,2-dichloroethylene. A methanotrophic culture was prepared in the first stage and chlorinated organic solvents was degraded in the second stage. Microorganism growth in the biofilms and biofilm properties have been studied for a few decades [13,14]. Biofilm is a multicomponent structure consisting of live microorganisms and extracellular polymeric substances (EPS). EPS are natural polymers secreted by the microbial cells into their surrounding environment and facilitate attachment of the cells to solid surfaces [15]. The main application of the biofilm engineering is concerned with designing new types of bioreactors including the membrane bioreactor, fluidized bed bioreactor, and biofilters. Biofilms have positive influences on the characteristics of bioreactors including high cell concentration, high resistance to inhibition resulting from the toxins, prevention of the cell washout, and possibility to separate liquid and cell residence times [16,17]. Owing to the advantages of biofilms, many experimental studies and mathematical modeling have been conducted in the last decades to acquire knowledge about the technology of bioreactors with the biofilm layer. Sevillano et al. [18] utilized cyclodextrin polymer (CDP) particles, which synthesized by crosslinking *β*-cyclodextrin with epichlorohydrin, as biofilm attachment media and then loaded in a fluidized bed bioreactor to treat phenol from wastewater.

Two main approaches, i.e., analytical and numerical, have been proposed in the literature for simulation of biofilms [19]. The former has significant limitations, e.g., considering a process with multi-substrate kinetics is not possible and it can only be used for zero- and first-order kinetics [20]. Skoneczny and Tabiś [21] proposed a model to determine the steady-states of a tubular bioreactor. They used orthogonal collocation method to solve mathematical model. It was found that changes in bioreactor diameter strongly affect degree of conversion of the substrate. Skoneczny and Cioch [22] simulated a bioreactor in steady-state conditions. They considered axial dispersion of liquid phase in the modeling and examined the deviation from an ideal plug flow. It was found that, for Peclet numbers above 40, plug flow of liquid could be assumed. Skoneczny and Cioch [23] studied CSTBR and tubular bioreactor with the biofilm on inner wall. They applied orthogonal collocation on finite elements (OCFE) to numerically solve the mathematical equations. The accuracy of OCFE was compared with the results obtained using shooting method. They also determined steady-state distribution of the substrate concentration inside the bioreactor.

Xu et al. [24] dissolved zeolitic imidazolate framework-8 (ZIF-8) nanoparticles in Brostol's solution to prepare ZIF8-SE medium, and then it was used in a horizontal tubular photobioreactor to enhance the CO_2_ absorption by microalgal cultivation. They found that mass transfer of CO_2_ and its concentration in the medium can be improved as a result of ZIF-8 particles. Sriputorn et al. [25] coupled a CSTRB and two tubular bioreactors to increase the efficiency of ethanol production from sweet sorghum stem juice in a very high gravity fermentation. The semi-continuous solid-state fermentation in a tubular bioreactor was studied by Wang et al. [26] and the bioreactor performance were compared with a batch process in terms of the degrees of outlet CO_2_ and starch degradation. A modeling for microbial aerobic process in a bubble column bioreactor with biofilm layer on inner wall was proposed by Skoneczny and Cioch-Skoneczny [27]. They studied the effect of biofilm presence on bioreactor steady-states using two mathematical algorithms and the results were compared.

As stated in the literature [1,28], modeling and theoretical studies of tubular bioreactors are requisite and essential to optimize the design and operational conditions. Investigations show that most of the researchers considered simplifying assumptions in the simulations, e.g., detachment of biomass from the biofilm and interphase mass transfer resistance of biofilm and liquid were neglected [21,29]. To the best of our knowledge, all simulations studied the steady-state condition of tubular bioreactor and unsteady-state condition has not explored so far. To predict the bioreactor behavior using simulation, steady- and unsteady-state approaches have benefits and difficulties. Depending on the specific problem at hand, the suitable approach should be chosen. Being more facile and efficient are the merits of steady-state method; however, it can't capture transient effects. Although unsteady-state method is more complicated and computationally intensive, it is capable of providing a deep understanding about the dynamic behavior of bioreactors.

The objective of this work is to examine the anaerobic biodegradation process in a tubular bioreactor with biofilm layer for steady- and unsteady-state operations. For a realistic modeling, biofilm was considered to be composed of active and inactive layers, and biomass detachment and mass transfer between biofilm and liquid were taken into account. In addition, a proportional-integral (PI) controller was tuned using genetic algorithm to regulate the degree of conversion of substrate for servo and regulatory modes. To check the validity of modeling, simulation results for substrate concentration at steady-state condition were compared with experimental data reported in Ref. [30].

## 2 Governing equations for tubular bioreactor

In this work, mathematical modeling of a tubular bioreactor with a homogeneous biofilm layer is conducted and anaerobic microbiological degradation of glucose as substrate is studied. The liquid phase is introduced from one end of the bioreactor and substrate diffuses radially from liquid to the biofilm layer and is consumed by the biomass. During mass transfer between the liquid and biofilm, biomass detachment from biofilm may occur; therefore, continuous phase passing through the bioreactor contains biomass as well. For a differential volume of bioreactor, unsteady-state mass balance equations for substrate A and biomass B are as follows [21]:

(1a) $$\frac{\partial C_{A}^{c}}{\partial t}=-u\frac{\partial C_{A}^{c}}{\partial x}-r_{A}^{c}\left(C_{A}^{c},C_{B}^{c}\right)\;-\frac{4k_{sA}\zeta }{d_{rb}}\;\left(C_{A}^{c}-C_{As}\right)$$

(1b) $$\frac{\partial C_{B}^{c}}{\partial t}=-u\frac{\partial C_{B}^{c}}{\partial x}+r_{B}^{c}\left(C_{A}^{c},C_{B}^{c}\right)+\frac{4}{d_{rb}}\;r_{\det}$$

Where C is the mass concentration, u is the liquid velocity, $k_{sA}$ is the mass transfer coefficient of substrate between biofilm and liquid phase, ζ is the degree of surface development, $d_{rb}$ is the inner diameter of bioreactor with biofilm layer, and $r_{\det}$ is the detachment rate of biomass from biofilm. The superscript c and subscript s denote continuous phase and surface of biofilm, respectively. The left hand side (LHS) term of Eq. (1a), (1b) is accumulation and the first term in right hand side (RHS) is the convective mass flow. The second term in RHS of Eq. (1a) is the consumption rate of substrate via free biomass in liquid phase and that of Eq. (1b) is the growth rate of free biomass. The third term in RHS of Eq. (1a) is the mass transfer rate of substrate from liquid to the biofilm and that of Eq. (1b) is the detachment rate of active biomass from biofilm.

The boundary conditions related to Eq. (1a), (1b) are expressed as follows [21]:

(2a) $$C_{A}^{c}\left(x=0,t\right)=C_{A}^{f}$$

(2b) $$C_{B}^{c}\left(x=0,t\right)=0$$

where $C_{A}^{f}$ is the substrate concentration in inlet feed stream. The detachment rate of biomass from biofilm layer, $r_{\det}$, can be calculated from [21]

(3) $$r_{\det}=X_{B}L_{a}{\bar{r}}_{B}^{b},0\leq X_{B}<1$$

where $X_{B}$ is the fraction of active biomass transferred from bofilm to the liquid phase, $L_{a}$ is the thickness of active biofilm, and ${\bar{r}}_{B}^{b}$ is the average growth rate of biomass in biofilm and can be calculated from [21]

(4) $${\bar{r}}_{B}^{b}=\frac{1}{L_{a}}\int_{0}^{L_{a}}r_{B}^{b}\left[C_{A}^{b}\left(x\right)\right]dx$$

The substrate A is transferred from liquid phase to the surface of biofilm layer and then transported by diffusion mechanism through the biofilm and is consumed by microbial cells. The unsteady-state mass balance for substrate A in the biofilm layer can be described by the following equation [21]:

(5) $$\frac{{\partial C}_{A}^{b}}{\partial t}=\frac{D_{eA}}{r}\left[\frac{{\partial C}_{A}^{b}}{\partial r}+r\frac{\partial^{2}C_{A}^{b}}{{\partial r}^{2}}\right]-r_{A}^{b}\left(C_{A}^{b}\right)$$

where $D_{eA}$ and $r_{A}^{b}$ are the diffusion coefficient and consumption rate of substrate A in biofilm layer, respectively. The boundary conditions related to Eq. (5) can be expressed as follows [23]:

(6a) $$\frac{{\partial C}_{A}^{b}}{\partial r}{|}_{r=R}=0$$

(6b) $$D_{eA}\frac{\partial C_{A}^{b}}{\partial r}{|}_{r=R-L_{a}}=k_{sA}\left[{\bar{C}}_{A}^{\;c}-C_{A}^{b}\left(L_{a}\right)\right]$$

where ${\bar{C}}_{A}^{\;c}$ and R are the average concentration of substrate A in liquid and radius of bioreactor with biofilm layer, respectively.

To numerically solve Eq. (1a), (1b) and (5) for the steady-state and unsteady-state conditions, a simpler method is to use dimensionless variables. Here, dimensionless variables of α, β, η, Z, and z are defined as follows [23]:

(7) $$\alpha =\frac{C_{A}^{f}-C_{A}^{c}}{C_{A}^{f}},\beta =\frac{C_{B}^{c}}{C_{A}^{f}},\eta =\frac{C_{A}^{b}}{C_{A}^{c}},Z=\frac{x}{L_{r}},z=\frac{R-r}{L_{a}}$$

where x and r are the coordinates in axial and radial directions, respectively, and $L_{r}$ is the length of bioreactor. Hence, dimensionless forms of Eqs. (1a), (1b), (5) can be expressed by the following equations [23]:

(8a) $$\tau^{c}\frac{\partial \alpha }{\partial t}=-\frac{\partial \alpha }{\partial Z}+\frac{\tau^{c}}{C_{A}^{f}}∙r_{A}^{c}\left(\alpha ,\beta \right)+\frac{4\tau^{c}k_{sA}\zeta }{d_{rb}}\left(1-\alpha \right)\left(1-\eta \left(1,t\right)\right)$$

(8b) $$\tau^{c}\frac{\partial \beta }{\partial t}=-\frac{\partial \beta }{\partial Z}+\frac{\tau^{c}}{C_{A}^{f}}∙r_{B}^{c}\left(\alpha ,\beta \right)+\frac{4\tau^{c}}{d_{rb}}∙\frac{r_{\det}}{C_{A}^{f}}$$

(9) $$\frac{\partial \eta }{\partial t}=\frac{D_{eA}}{L_{a}^{2}}\;\frac{\partial^{2}\eta }{\partial z^{2}}+\frac{D_{eA}}{L_{a}\left(L_{a}z-R\right)}\frac{\partial \eta }{\partial z}-\frac{r_{A}^{b}\left(\eta \right)\;}{C_{A}^{c}}$$

where $\tau^{c}$ is the residence time of liquid phase in bioreactor and is defined as $\tau^{c}=L_{r}/u$.

The following boundary conditions are associated with Eqs. (8a), (8b), (9) [23]:

(10a) α(Z=0,t)=0

(10b) β(Z=0,t)=0

(11a) $$\frac{\partial \eta }{\partial z}{|}_{z=0}=0$$

(11b) $$\frac{\partial \eta }{\partial z}{|}_{z=1}={Bi}_{A}\left(1-\eta \left(1,t\right)\right)$$

where ${Bi}_{A}$ is the Biot number for substrate A and is defined as ${Bi}_{A}=\frac{k_{sA}L_{a}}{D_{eA}}$.

In this study, glucose is chosen as the carbonaceous substrate and its biodegradation via *Pseudomonas aeruginosa* is simulated. Beyenal and Lewandowski [31] proposed a kinetic model for this microbiological process. In studying biofilm qualities, *P. aeruginosa* is oftentimes used, maybe it is due to the abundant researches conducted by geneticists and the biokinetic parameters for microbial growth is well known [31]. Based on this work, biomass growth rate and consumption rate of the substrate in liquid phase and biofilm layer can be given by the following relations:

(12a) $$r_{B}^{c}\left(C_{A}^{c},C_{B}^{c}\right)=\frac{kC_{A}^{c}}{K_{A}+C_{A}^{c}}∙C_{B}^{c}$$

(12b) $$r_{A}^{c}\left(C_{A}^{c},C_{B}^{c}\right)=\frac{1}{W_{BA}}∙r_{B}^{c}\left(C_{A}^{c},C_{B}^{c}\right)$$

(13a) $$r_{B}^{b}\left(C_{A}^{b}\right)=\frac{kC_{A}^{b}}{K_{A}+C_{A}^{b}}∙\rho_{a}$$

(13b) $$r_{A}^{b}\left(C_{A}^{b}\right)=\frac{1}{W_{BA}}∙r_{B}^{b}\left(C_{A}^{b}\right)$$

where k is the maximum specific growth rate, $K_{A}$ is the saturation constant for substrate A, $W_{BA}$ is the biomass-to-substrate yield coefficient, and $\rho_{a}$ is the concentration of active biomass in biofilm.

## 3 Numerical methods for solving the governing equations

A code has been developed using MATLAB software to simulate the behavior of tubular bioreactor with a biofilm layer for two scenarios of steady- and unsteady-state. For steady-state conditions, LHS of Eqs. (8a), (8b), (9) is set to zero and then the non-linear differential equations and boundary conditions expressed in Eqs. (10b), (10a), (11b), (11a) are concurrently solved using finite difference method. Differential equations of (8a), (8b) are initial value problem while differential equation of (9) is a boundary value problem and the BVP4C code of MATLAB is implemented to solve it. For unsteady-state conditions, the explicit method is applied for the temporal derivatives of Eqs. (8a), (8b), (9), whereas the finite difference method is employed for the spatial derivatives. The boundary conditions are considered the same as Eq. (10b), (10a) and (11b), (11a). In addition, distribution concentration of the substrate and biomass in liquid phase and biofilm layer, obtained from the steady-state solution of the governing equations, are set as initial conditions.

## 4 Results and discussions

To simulate a tubular bioreactor with a biofilm layer, governing equations explained in section 2 are solved together. To implement the simulations, bioreactor diameter and substrate concentration in the inlet feed are needed. Other parameters regarding the diffusion and mass transfer coefficients of substrate, and kinetic parameters for calculating biomass growth and substrate consumption rates are also required. The values of the constant parameters applied in simulations are summarized in Table 1, where the empirical parameters are taken from Ref. [21]. The simulation results obtained from this research are presented and discussed in four sections. In the first section, steady-state concentration of the substrate in a continuous stirred tank bioreactor is predicted and compared with experimental data reported in the literature [30]. The second section is devoted to the results obtained from steady-state simulation of tubular bioreactor and the effects of simulation parameters on performance of the bioreactor are studied, whereas the results regarding unsteady-state simulation are presented in the third section. The fourth section presents the design of a controller to regulate the substrate concentration in liquid when a change in set point or a disturbance occurred in the system.

**Table 1:** The values of constant parameters used in the simulations [21].

Parameter	Value
$C_{A}^{f}$, kg/m^3^	0.1
$d_{rb}$, m	0.1
$D_{eA}$, m^2^/hr	6.01 × 10^−7^
k, 1/hr	0.32
$k_{sA}$, m/hr	0.18
$K_{A}$, kg/m^3^	0.0203
$L_{a}$, μm	90
$W_{BA}$, kg B/kg A	0.628
$X_{B}$	0.5
ζ	2
$\rho_{a}$, kg/m^3^	60

### 4.1 Validation of simulation results

**Fig. 1:**
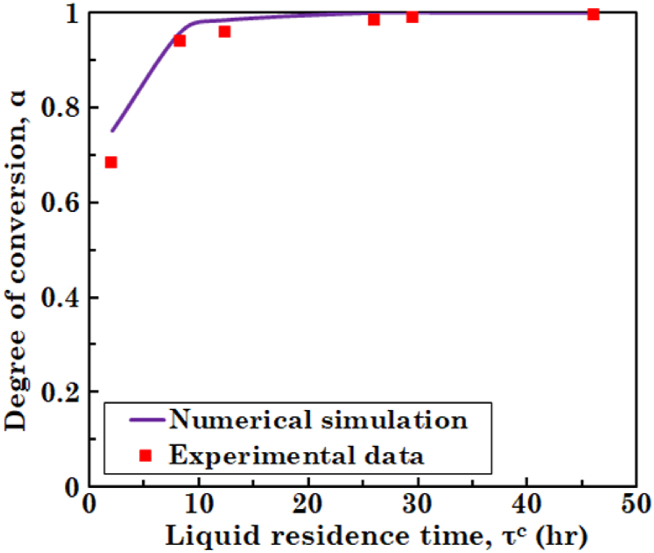
Comparison of simulation results with experimental data taken from Ref. [30] for degree of conversion of the substrate in liquid phase at steady-state condition.

To verify the resulting outcomes of numerical simulation, validation against experimental data is necessary. Fig. 1 shows the simulation results and experimental data taken from Ref. [30]. Dokianakis et al. [30] obtained steady-state concentration of the substrate in a CSTBR for various residence time of the liquid. Due to lack of the experimental data regarding tubular bioreactors with a biofilm layer, validation is carried out with the experimental data of a CSTBR. As it can be seen in Fig. 1, degree of conversion of the substrate at various residence time of liquid, predicted by the numerical simulation, is in well agreement with experimental data. Calculating relative error for simulation results of Fig. 1 suggests that almost the entire relative errors are less than 5 % and the only exception is the degree of conversion at $\tau^{c}=2.1\;h$, which is about 10 %.

### 4.2 Steady-state simulation

**Fig. 2:**
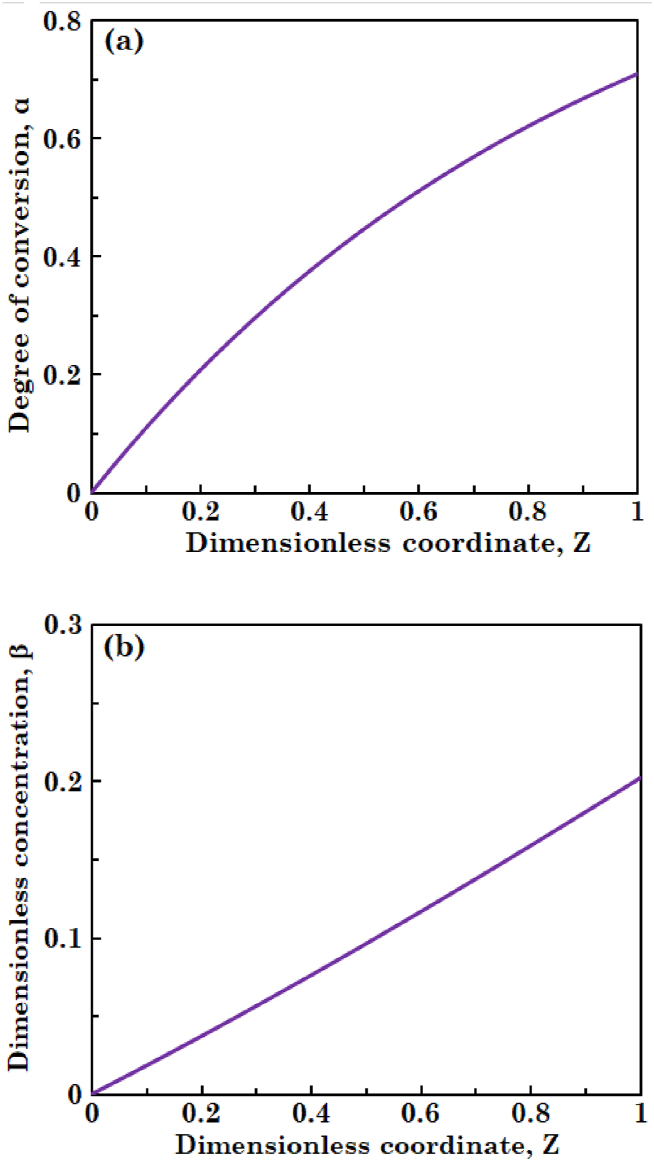
The profiles of (a) degree of conversion of substrate and (b) dimensionless concentration of biomass in liquid phase at steady-state condition.

**Fig. 3:**
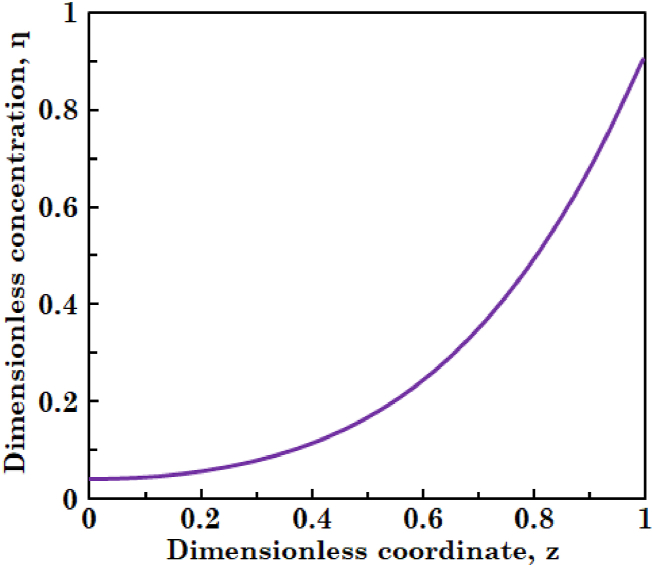
The profile of dimensionless concentration of substrate in biofilm layer at steady-state condition.

In this section, governing equations are solved in steady-state mode and the concentrations of substrate and biomass in liquid phase and that of substrate in biofilm layer are obtained. Fig. 2(a) and (b) illustrate variations in degree of conversion of the substrate and dimensionless concentration of the biomass in liquid along the bioreactor, respectively. It can be observed in Fig. 2(a) that degree of conversion of the substrate increases. As liquid phase moves along the bioreactor, more and more substrate penetrate into the biofilm and is consumed by biomass. Fig. 2(b) shows that biomass concentration in liquid phase also increases. This is due to the detachment phenomenon of biomass from biofilm and biomass growth occurred in the liquid phase. Fig. 3 indicates the profile of dimensionless concentration of substrate across the biofilm layer. It is obvious from this figure the highest substrate concentration is obtained at biofilm surface and the substrate concentration decreases across the biofilm layer. This is because of consumption of substrate molecules by the microorganisms during diffusion through the biofilm.

#### 4.2.1 Influence of bioreactor diameter

**Fig. 4:**
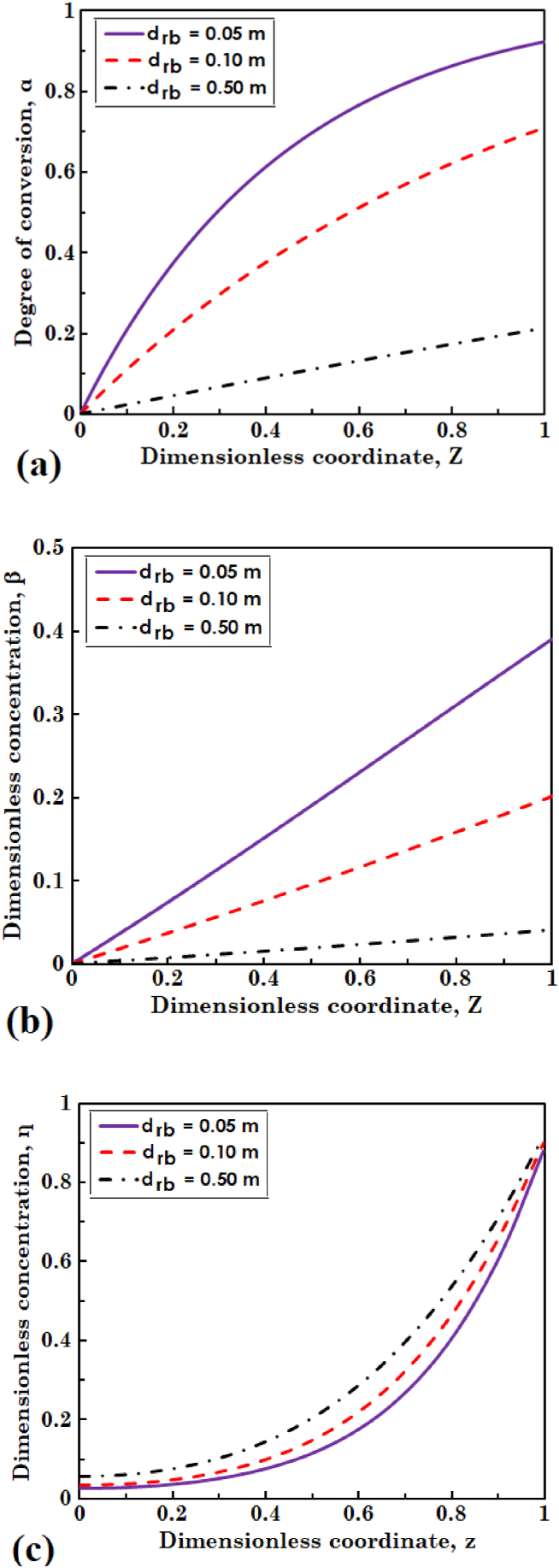
The effect of bioreactor diameter on (a) degree of conversion of substrate and (b) dimensionless concentration of biomass in liquid phase, and (c) dimensionless concentration of substrate in biofilm layer.

The influence of bioreactor diameter on distribution of the degree of conversion of substrate and dimensionless concentration of biomass in liquid, and dimensionless concentration of substrate in the biofilm is shown in Fig. 4 (a), (b), and (c), respectively. In this section, three values of 0.05, 0.1, and 0.5 m are considered for the diameter of bioreactor and the other parameters were kept unchanged. It is clear from Fig. 4(a) lowering the bioreactor diameter results in increasing degree of conversion of the substrate. The flow rate of entering feed is decreased as the diameter of bioreactor lowers, because residence time of the liquid phase remains unchanged. This means that the amount of substrate entering with inlet feed is reduced and substrate molecules can diffuse more easily into the biofilm. On the other hand, because the specific surface area of the biofilm defined as $a_{s}=4\zeta /d_{rb}$ is proportional to the inverse of bioreactor diameter, surface area of the biofilm that is available to substrate increases with a drop in the diameter of bioreactor. With an increase of biofilm surface area and easier penetration of substrate into the biofilm, degree of conversion of the substrate goes up.

It can also be observed in Fig. 4(b) that dimensionless concentration of the biomass in liquid phase goes up with decreased bioreactor diameter. According to Eq. (8b), this is because of increasing amount of the biomass detached from biofilm. Unlike variations of α and β observed in Fig. 4(a) and (b), Fig. 4(c) shows that dimensionless concentration of the substrate in biofilm is reduced with lowering bioreactor diameter. The reason is that lower concentration distribution of the substrate in liquid phase along the bioreactor drops the mass transfer rate of substrate into the biofilm layer, which in turn leads to a lower concentration profile of the substrate in biofilm. The results acquired in Fig. 4(a) and (b) are in good agreement with the study of Skoneczny and Tabiś [21].

#### 4.2.2 Influence of fraction of active biomass transferred to the liquid phase

**Fig. 5:**
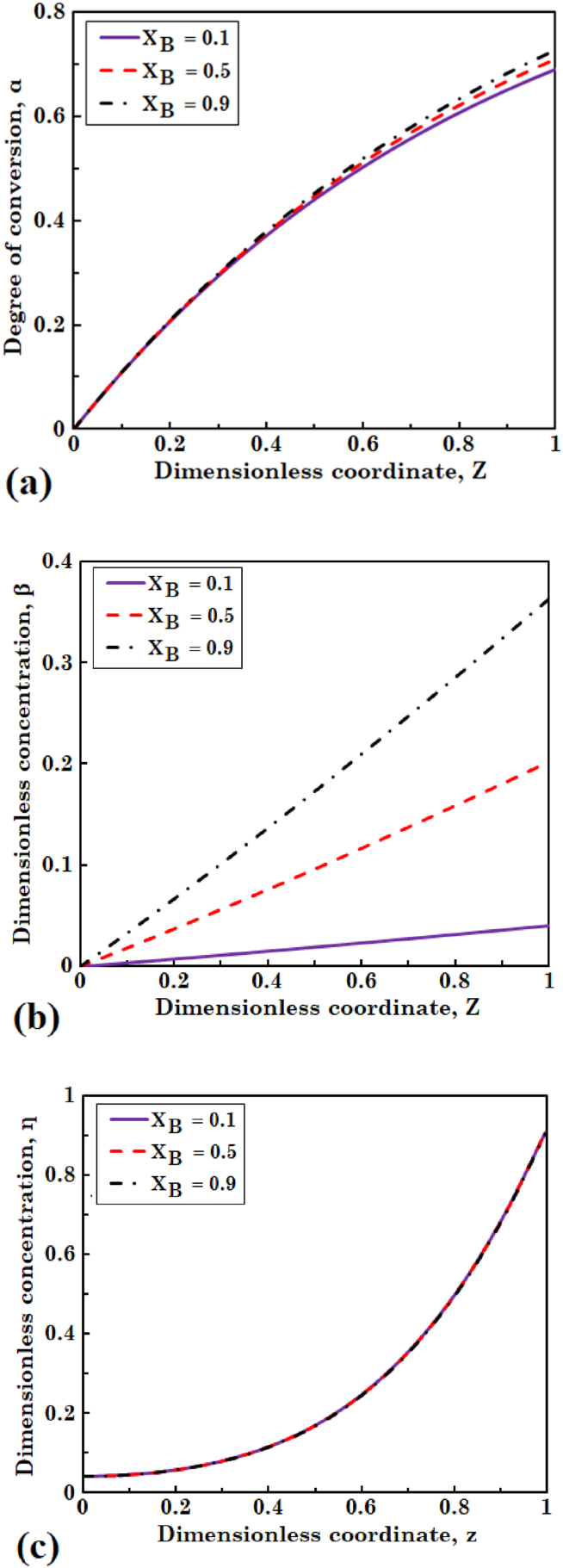
The effect of fraction of active biomass transferred to the liquid phase on (a) degree of conversion of substrate and (b) dimensionless concentration of biomass in liquid phase, and (c) dimensionless concentration of substrate in biofilm layer.

In this section, three values of 0.1, 0.5, and 0.9 are considered for fraction of active biomass transferred from biofilm to the liquid phase ($X_{B}$), to explore the effect on variations of biomass and substrate concentrations. Fig. 5(a) and (b) illustrate the profiles of degree of conversion of the substrate and dimensionless concentration of the biomass in liquid phase, respectively. Fig. 5(c) shows variations in dimensionless concentration of the substrate across the biofilm layer. As it can be observed in Fig. 5(a) and (c), concentrations of the substrate in both biofilm and liquid phase insignificantly change. It indicates that almost all the substrate consumed in bioreactor is because of the diffusion phenomenon of substrate into the biofilm and free biomass in the liquid phase plays a small role in consuming the substrate. Olivieri et al. [32] carried out a simulation study about the dynamic behavior of granular-supported biofilm in a mixed three-phase bioreactor. The little effect of the parameter $X_{B}$ on substrate conversion agreed with the results obtained by Olivieri et al. [32] for moderate to high dilution rate. However, as can be seen in Fig. 5(b), concentration of the biomass in liquid is boosted with an increase in the value of $X_{B}$. This is due to increased detachment rate of the biomass from biofilm with increasing $X_{B}$. Skoneczny and Tabiś [21] reported similar results for the influence of the parameter $X_{B}$ on concentrations of the substrate and biomass in liquid phase.

#### 4.2.3 Influence of residence time of the liquid phase

**Fig. 6:**
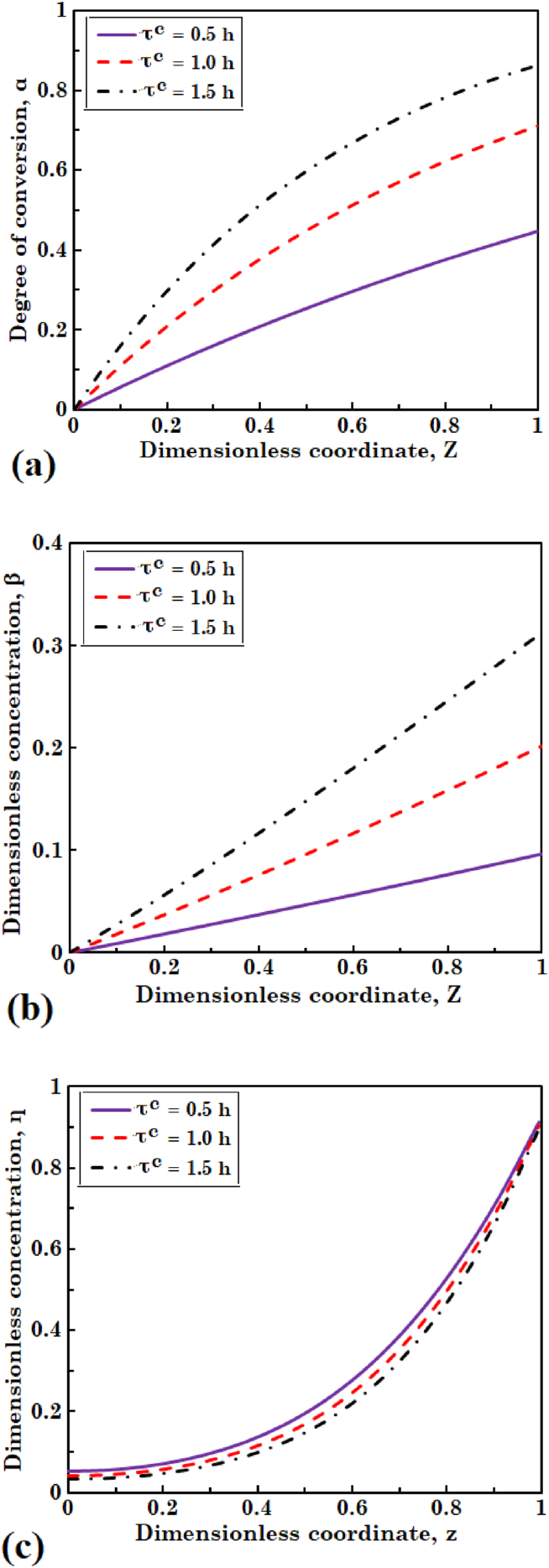
The effect of residence time of liquid on (a) degree of conversion of substrate and (b) dimensionless concentration of biomass in liquid phase, and (c) dimensionless concentration of substrate in biofilm layer.

To investigate the effect of residence time of the liquid phase on distribution of the biomass and substrate concentrations, three values of 0.5, 1.0, and 1.5 h are considered. Fig. 6(a), (b), and (c) display degree of conversion of the substrate and dimensionless concentration of biomass in liquid, and dimensionless concentration of the substrate in biofilm, respectively. As residence time of the liquid phase in bioreactor goes up, more substrate can diffuse into the biofilm; as a result, degree of conversion of the substrate increases. The results of Fig. 6(b) indicate that biomass concentration in the liquid phase rises with an increase of residence time of the continuous phase. As liquid resides a further time in the bioreactor, contact period of the liquid and biomass goes up. Consequently, amount of the biomass detached from biofilm and substrate consumption due to the reaction with free biomass increase. Dokianakis et al. [30] investigated the aerobic biodegradation of nitrite in a CSTR with biofilm layer. Ivancic et al. [33] and Rezic et al. [34] examined the fermentative glucose conversion in a horizontal rotating tubular bioreactor having O-ring shaped partition walls. The findings of these studies regarding the effects of liquid residence time on degree of conversion of substrate and biomass concentration are in good agreement with the findings of this research. It is evident from Fig. 6(c) that increasing residence time of the liquid causes a small decrease of the substrate concentration in biofilm. Because of diminishing concentration distribution of the substrate along the bioreactor, observed in Fig. 6(a), mass transfer rate of substrate into biofilm decreases; consequently, substrate concentration in biofilm drops.

### 4.3 Unsteady-state simulation

**Fig. 7:**
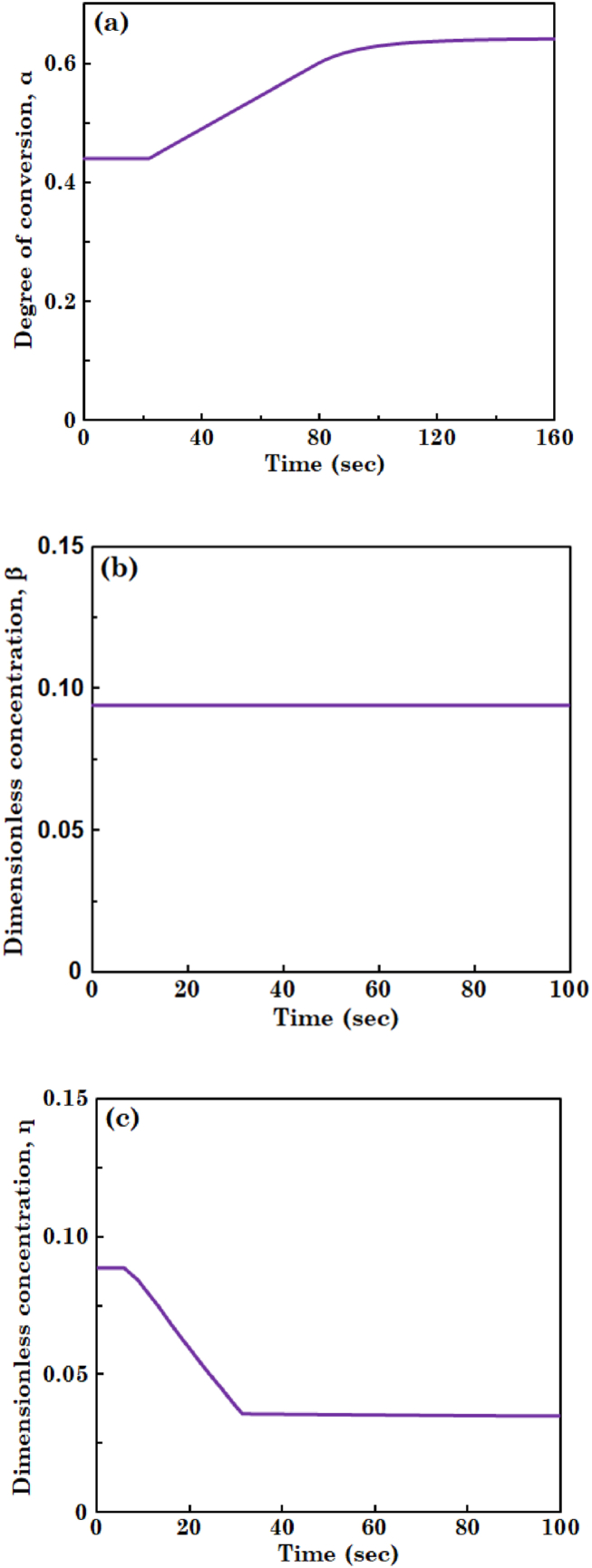
The profiles of (a) degree of conversion of substrate and (b) dimensionless concentration of biomass in liquid phase, and (c) dimensionless concentration of substrate in biofilm layer at unsteady-state condition.

In this section, it is considered that tubular bioreactor working at steady-state condition undergoes a reduction of 20 % in inlet concentration of the substrate. The inlet concentration of substrate is changed as a step function at time equal to zero and variations in the concentrations of substrate and biomass in the liquid and biofilm are explored over time. Fig. 7(a), (b), and (c) illustrate degree of conversion of the substrate and dimensionless concentration of the biomass in liquid, and dimensionless concentration of the substrate in biofilm, respectively. In these figures, concentrations are plotted versus time at dimensionless axial and radial positions (Z and z) equal to 0.5 and 0.4, respectively.

It can be observed from Fig. 7(a) and (c) that degree of conversion and dimensionless concentration of the substrate in liquid and biofilm remains unchanged for nearly 22 s and 5 s, respectively. These time periods show the delay time in concentration profiles of the substrate, then degree of conversion of the substrate increases and dimensionless concentration of the substrate decreases. With a decrease in inlet concentration of the substrate, second term on right hand side of Eq. (8a) goes up, which in turn leads to a rise of degree of conversion of the substrate in Fig. 7(a). Reducing concentration of substrate along the bioreactor results in decreasing driving force for mass transfer of the substrate into the biofilm. Therefore, as indicated in Fig. 7(c), dimensionless concentration of the substrate in biofilm drops. It can also be seen in these two figures degree of conversion of the substrate reaches a new steady-state concentration after 110 s while dimensionless concentration of the substrate reaches after 30 s Fig. 7(b) shows that dimensionless concentration of the biomass in liquid phase remains constant. Diminishing inlet concentration of the substrate causes the second and third terms in RHS of Eq. (8b) increases; however, magnitude of these terms is much smaller than that of the first term. Accordingly, biomass concentration in this figure stays constant.

### 4.4 Designing a proportional-integral controller

To treat wastewater containing a toxic substance like phenol as substrate using a tubular bioreactor, it is necessary to maintain the concentration of substrate at a certain level in liquid exiting bioreactor. In this regard, to regulate concentration of the substrate in tubular bioreactor, a proportional-integral (PI) controller is designed. Here, degree of conversion of the substrate is considered as the controlled variable and inlet concentration of the substrate is assumed to be the manipulated variable. A first-order plus time-delay transfer function according to the following equation is applied for the process [35].

(14) $$G^{p}\left(s\right)=\frac{G_{s}}{\tau s+1}e^{-\tau_{D}s}$$

where $G_{s}$, $\tau_{D}$, τ, and s are the gain, time delay, time constant, and Laplace variable, respectively. Based on the results shown for the degree of conversion at unsteady-state condition, values of the parameters of the transfer function are obtained as $G_{s}=-10$, $\tau_{D}=21\;s$, and τ=46s.

**Fig. 8:**
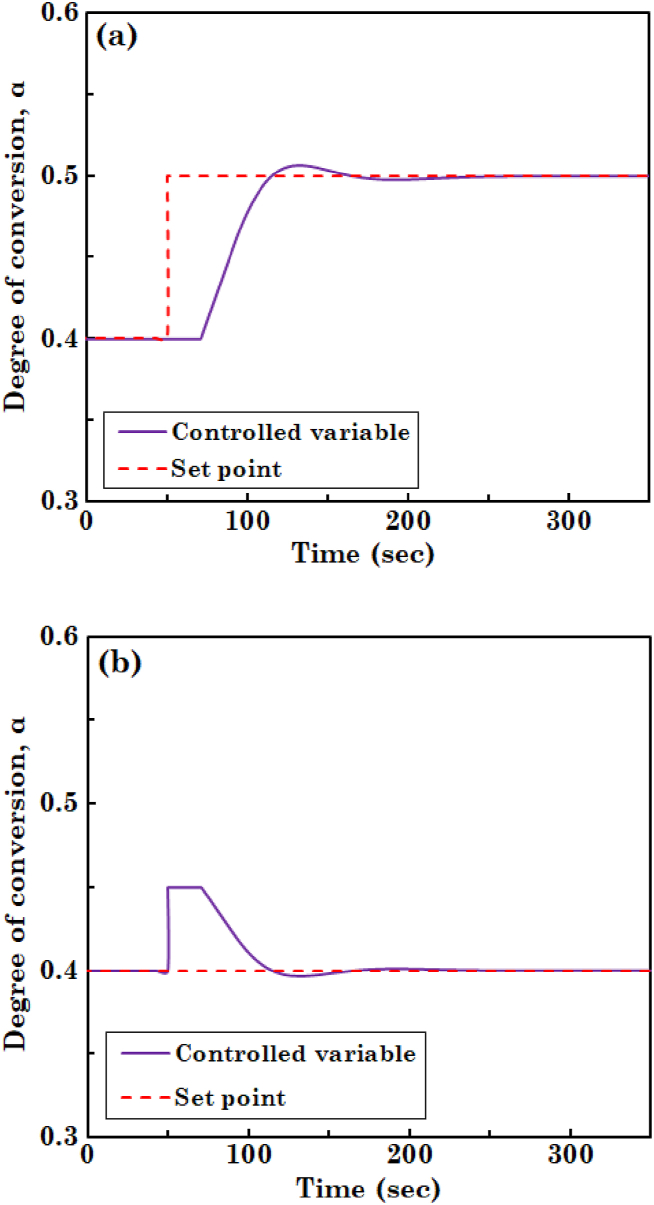
Set-point tracking performance of a PI controller for (a) servo control and (b) regulatory control.

To analyze the response of the system for a change in the value of degree of conversion of the substrate, transfer function of the PI controller, i.e., $G_{c}\left(s\right)=K_{P}+\frac{K_{I}}{s}$, is defined in the Simulink environment of MATLAB software. The constant parameters of the controller, i.e., $K_{P}$ and $K_{I}$, are tuned via the genetic algorithm and the values are as follows: $K_{P}=-0.131$ and $K_{I}=0.02$. After introducing transfer functions of the process and PI controller, response of the control system to a disturbance and set-point change is probed. Fig. 8(a) and (b) depict the performance of set-point tracking of the PI controller for servo control, i.e., response to set-point change, and regulatory control, i.e., disturbance rejection, respectively. As it is obvious in these figures, PI controller reacts to variation in set point and disturbance occurred in the system with a lag time of nearly 20 s and can regulate the controlled variable, i.e., degree of conversion of the substrate, within 120 s. It can also be observed in Fig. 8(a) and (b) that response of the control system has low and desirable overshoot and undershoot, respectively.

## 5 Conclusions

A tubular bioreactor with biofilm was considered and anaerobic biodegradation of substrate was investigated. In this regard, the effects of parameters including bioreactor diameter, fraction of active biomass transferred to liquid phase, and liquid residence time were explored. Simulations were carried out for steady-state and unsteady-state conditions and a proportional-integral (PI) controller was tuned to control the degree of conversion of substrate in liquid phase. Based on the results obtained, the following conclusions can be drawn.

a) Decreasing diameter of the bioreactor leads to an increase in the degree of conversion of substrate, whereas dimensionless concentration of the substrate in biofilm lowers.
b) Variations in fraction of active biomass transferred to liquid phase have insignificant effect on distribution of the substrate concentrations in both liquid and biofilm phases.
c) Dimensionless concentration of biomass in liquid phase increases with an increase in the fraction of active biomass transferred to liquid.
d) As residence time of the liquid rises, degree of conversion of the substrate is boosted while a small decrease of substrate concentration in the biofilm layer is observed.
e) Dimensionless concentration of the biomass in liquid goes up with decreasing bioreactor diameter and increasing liquid residence time.
f) A PI controller is designed and its constant parameters are tuned using genetic algorithm. The values of controller parameters are obtained as $K_{P}=-0.131$ and $K_{I}=0.02$.
g) The performance of the controller for servo mode, i.e., response to set-point change, and regulatory mode, i.e., disturbance rejection, is explored and observed that controller can well regulate degree of conversion of the substrate in liquid.

**Table:** Nomenclature

$a_{s}$	specific surface area of biofilm, m^−1^
${Bi}_{A}$	Biot number for substrate A
C	mass concentration, kg m^−3^
$d_{rb}$	inner diameter of bioreactor with biofilm layer, m
$D_{eA}$	diffusion coefficient of substrate A in biofilm, m^2^ h^−1^
$G_{c}$	transfer function of the controller
$G_{s}$	gain of control system
$G^{p}$	transfer function of the process
k	maximum specific growth rate, h^−1^
$k_{sA}$	mass transfer coefficient of substrate between biofilm and liquid phase, m h^−1^
$K_{A}$	saturation constant for substrate A, kg m^−3^
$K_{I}$	integral constant of the controller
$K_{P}$	proportionality constant of the controller
$L_{a}$	active biofilm thickness, m
$L_{r}$	bioreactor length, m
r	coordinate in the radial direction of bioreactor, m
$r_{A}$	consumption rate of substrate A, kg m^−3^ h^−1^
$r_{B}$	biomass growth rate, kg m^−3^ h^−1^
$r_{\det}$	detachment rate of biomass from biofilm, kg m^−2^ h^−1^
R	radius of bioreactor with biofilm layer, m
t	time, h^−1^
u	liquid velocity, m h^−1^
$W_{BA}$	yield coefficient of biomass to substrate A
x	coordinate in the axial direction of bioreactor, m
$X_{B}$	fraction of active biomass transferred from biofilm to the liquid phase
z	dimensionless radial direction for biofilm
Z	dimensionless axial direction in bioreactor
Greek Symbols	
α	degree of conversion of the substrate A in liquid phase
β	dimensionless concentration of biomass in liquid phase
ζ	degree of surface development
η	dimensionless concentration of substrate A in biofilm
$\rho_{a}$	concentration of active biomass in biofilm, kg m^−3^
τ	time constant of the control system, s
$\tau^{c}$	residence time of the liquid phase in bioreactor, h
$\tau_{D}$	time delay of the control system, s
Subscripts/Superscripts	
A	carbonaceous substrate
b	biofilm
B	Biomass
c	continuous phase (liquid)
f	inlet feed stream
s	surface of biofilm

## Data availability statement

Data will be made available on request.

## CRediT authorship contribution statement

**Saman Malekahmadi:** Software, Investigation, Conceptualization. **Amirhossein Yousefnezhadazizi:** Validation, Software, Conceptualization. **Hossein Askaripour:** Writing – review & editing, Writing – original draft, Validation, Supervision.

## Declaration of competing interest

The authors declare that they have no known competing financial interests or personal relationships that could have appeared to influence the work reported in this paper.
